# Total baseline tumor size predicts survival among patients with advanced small-cell lung cancer receiving chemotherapy plus programmed death-ligand 1 inhibitor as first-line therapy: a multicenter retrospective observational study

**DOI:** 10.3389/fonc.2024.1400277

**Published:** 2024-11-04

**Authors:** Anna Tanaka, Shuhei Teranishi, Yukihito Kajita, Tomofumi Hirose, Ayami Kaneko, Yu Sairenji, Hidetoshi Kawashima, Kentaro Yumoto, Toshinori Tsukahara, Kenji Miura, Nobuaki Kobayashi, Masaki Yamamoto, Ryuichi Nishihira, Makoto Kudo, Naoki Miyazawa, Masanori Nishikawa, Takeshi Kaneko

**Affiliations:** ^1^ Respiratory Disease Center, Yokohama City University Medical Center, Yokohama, Japan; ^2^ Department of Pulmonology, Yokohama City University Graduate School of Medicine, Yokohama, Japan; ^3^ Department of Respiratory Medicine, Yokohama Sakae Kyosai Hospital, Yokohama, Japan; ^4^ Department of Respiratory Medicine, Kanto Rosai Hospital, Kawasaki, Japan; ^5^ Department of Respiratory Medicine, Yokohama Minami Kyosai Hospital, Yokohama, Japan; ^6^ Department of Respiratory Medicine, Chigasaki Municipal Hospital, Chigasaki, Japan; ^7^ Department of Respiratory Medicine, Yokohama Nanbu Hospital, Yokohama, Japan; ^8^ Department of Respiratory Medicine, Fujisawa Municipal Hospital, Fujisawa, Japan

**Keywords:** baseline tumor size, first-line therapy, immune checkpoint inhibitor, overall survival, programmed death-ligand 1 inhibitor, small-cell lung cancer

## Abstract

**Introduction:**

Total baseline tumor size (BTS) is a prognostic factor for programmed death 1 and programmed death-ligand 1 (PD-L1) inhibitor treatments. However, the prognostic value of total BTS for patients with small-cell lung cancer (SCLC) who receive chemotherapy plus PD-L1 inhibitor remains unknown. Thus, in this study, we aimed to determine whether total BTS is associated with prognosis in patients with SCLC who receive chemotherapy plus PD-L1 inhibitor as first-line therapy.

**Methods:**

This study included patients with extensive-stage SCLC or post-chemoradiotherapy recurrence of limited-stage SCLC who received chemotherapy plus PD-L1 inhibitor as first-line therapy from August 2019 to December 2022. The two lesions with the largest diameter among the measurable lesions in each organ were selected from up to five organs (maximum of 10 lesions), and the sum of all diameters was defined as total BTS. The patients were divided into two groups, large or small, with total BTS using X-tile software. Median survival was analyzed using the Kaplan–Meier method, and the groups were compared using the log-rank test. Univariate and multivariate analyses examined the association between total BTS and prognosis.

**Results:**

Fifty patients were included; 14% had large total BTS (>183.2 mm) and 86% had small total BTS (≤183.2 mm). The median observation period was 10.5 months. The large total BTS group showed significantly worse overall survival than the small total BTS group (median: 26.8 months vs. 5.7 months, *P* = 0.0003). The multivariate analysis indicated that large total BTS was an independent negative predictor of overall survival (hazard ratio: 7.14, 95% confidence interval: 1.89–26.96).

**Discussion:**

Total BTS is a potentially useful prognostic factor for patients with advanced SCLC who receive chemotherapy plus PD-L1 inhibitor as first-line therapy.

## 1 Introduction

Lung cancer is the leading cause of cancer-related death worldwide. Small-cell lung cancer (SCLC) comprises 15% of all lung cancer cases (1). Approximately two-thirds of SCLC cases already have extensive-stage (ES) disease at diagnosis, which progresses rapidly and has a poor prognosis (2, 3). However, for several decades, the standard therapy for ES-SCLC has not progressed beyond the administration of cytotoxic anticancer agents (4, 5). Recently, the phase III IMpower133 trial showed that adding the programmed death-ligand 1 (PD-L1) inhibitor, atezolizumab, to chemotherapy as the first-line therapy for ES-SCLC could improve the progression-free survival (PFS) and overall survival (OS) of patients (6). Similarly, the phase III CASPIAN trial indicated that adding durvalumab, a PD-L1 inhibitor, to chemotherapy as the first-line therapy for ES-SCLC could improve OS (7, 8). These results have now established the combination of chemotherapy and PD-L1 inhibitor as the first-line standard therapy for patients with ES-SCLC. However, compared to non-small-cell lung cancer (NSCLC), the effect of PD-L1 inhibitor is limited, and the prognosis for ES-SCLC remains poor (9–11).

Identification of predictors of chemotherapy plus PD-L1 inhibitor efficacy in ES-SCLC constitutes a pressing concern. Representative biomarkers for predicting response to programmed death 1 (PD-1)/PD-L1 inhibitors include PD-L1 expression and tumor mutation burden (12–14). However, neither of these biomarkers can identify patients with ES-SCLC who are likely to benefit from chemotherapy plus PD-L1 inhibitor (6, 15, 16). Prognostic factors that can be measured in real-world practice when patients with ES-SCLC undergo chemotherapy plus PD-L1 inhibitor include Eastern Cooperative Oncology Group Performance Status (ECOG PS) (17–19), brain metastases (18, 20), liver metastases (17, 19, 20), bone metastases (18, 19), peripheral blood neutrophil-to-lymphocyte ratio (NLR) (17, 21), and peripheral blood platelet-to-lymphocyte ratio (PLR) (22). However, none of these factors have been conclusively validated.

In NSCLC, head and neck cancer, and melanoma, the total baseline tumor size (total BTS), which is the sum of the diameters of measurable lesions before treatment begins, is reportedly a significant prognostic factor in patients receiving therapy, including PD-1/PD-L1 inhibitors (23–26). However, the association between total BTS and prognosis in patients with SCLC treated with chemotherapy and PD-L1 inhibitor has not yet been reported.

The aim of this retrospective, multicenter, observational study was to determine whether total BTS is associated with prognosis, particularly OS, the most critical endpoint in cancer treatment, in patients with SCLC who receive chemotherapy plus PD-L1 inhibitor as first-line therapy.

## 2 Materials and methods

### 2.1 Patients and data collection

This retrospective, multicenter, observational study was carried out in collaboration with five medical facilities in Kanagawa Prefecture, Japan. The study adhered to the principles of the Declaration of Helsinki and was approved by the Yokohama City University Ethics Board (approval number: B191200044). The waiver of informed consent was obtained for the study’s retrospective design. The study population included patients with SCLC who received chemotherapy plus PD-L1 inhibitor as the first-line therapy from August 2019 to December 2022. The patient eligibility criteria were as follows: (a) diagnosis of SCLC based on analysis of tissue or cell specimens; (b) diagnosis of ES-SCLC or post-chemoradiotherapy recurrence of limited-stage SCLC; and (c) use of carboplatin plus etoposide plus durvalumab, cisplatin plus etoposide plus durvalumab, or carboplatin plus etoposide plus atezolizumab as first-line therapy, between August 2019 and December 2022. We excluded patients who had tumors with a mixture of histological types other than small-cell carcinoma. We collected information on age, sex, history of smoking, ECOG PS, stage, presence of brain/liver/bone metastases, blood test data, and tumor size for each patient. A cutoff age of 65 years was used for analysis (6, 7). ECOG PS was categorized as good (score: 0–1) or poor (score: 2–4) (6, 7).

### 2.2 Hematological parameters

We collected data on absolute neutrophil count, absolute lymphocyte count (ALC), and platelet count (PLT) from blood tests on the day before or the day of starting first-line therapy. We defined NLR as the ratio of absolute neutrophil count to ALC and PLR as the ratio of PLT to ALC (27).

### 2.3 Evaluation of total BTS

BTS was measured using computed tomography of the chest, abdomen, and pelvis and magnetic resonance imaging of the brain before the start of first-line therapy. Measurable lesions were defined by the Response Evaluation Criteria in Solid Tumor (RECIST) version 1.1 (28), according to which non-lymph node lesions must have a long diameter of at least 10 mm, while lymph nodes must have a short diameter of at least 15 mm. The two lesions with the largest diameter among the measurable lesions in each organ were selected in up to five organs (a maximum of 10 lesions), and the sum of all diameters was defined as the total BTS (26).

### 2.4 Evaluation of treatment response

The tumor response was determined using the RECIST version 1.1. The objective response rate (ORR) was determined as the percentage of patients who achieved either a complete or partial response, and the disease control rate (DCR) was determined as the percentage of patients who achieved either a complete or partial response or stable disease. PFS was calculated as the time from the start of first-line therapy to disease progression, death, or the final follow-up (censored). OS was calculated as the time from the start of first-line therapy to death or the final follow-up (censored). Post-progression survival (PPS), which exhibits a stronger correlation with OS than PFS when chemotherapy is combined with a PD-L1 inhibitor as first-line therapy in patients with ES-SCLC, was also examined (29, 30). PPS was defined as the duration from the point of disease progression following first-line therapy until death or the last follow-up (censored). For PPS analysis, only patients whose first-line therapy resulted in PD were included. The follow-up period ended on August 6, 2023.

### 2.5 Statistical analysis

We identified cutoff values for NLR, PLR, and total BTS for OS, the most critical endpoint in cancer treatment, using X-tile 3.6.1 software (Yale University, New Haven, CT, USA). The X-tile 3.6.1 software sets cutoff values based on Kaplan–Meier log-rank chi-square values; it can demonstrate the robustness of the relationship between biomarkers and outcomes (31). Survival analysis for OS, PFS, and PPS was performed using the Kaplan–Meier method and assessed using the log-rank test. Moreover, the hazard ratio (HR) and its corresponding 95% confidence interval (CI) were determined by employing the Cox proportional hazards model. Univariate analysis was performed to determine the independent prognostic value of total BTS for OS, PFS, and PPS, and multivariate analysis was performed using only those factors for which *P <*0.05 was obtained in univariate analyses. Spearman’s rank correlation analysis and linear regression analysis were used to evaluate the correlation between PFS-OS and PPS-OS. Fisher’s exact test was used to compare categorical factors. Mann–Whitney *U*-test was used to compare the numerical data. Statistical significance was set at *P <*0.05, and all tests were two-sided. Statistical analyses were conducted using GraphPad Prism version 10 (GraphPad Software, San Diego, CA, USA) and EZR version 1.63 (Saitama Medical Centre, Jichi Medical University, Saitama, Japan) (32).

## 3 Results

### 3.1 Patient characteristics

The patient demographics are presented in **Table 1**, including data from 50 individuals. The median age was 72 years; two-thirds were male, and the vast majority had a history of smoking. More than three-quarters of patients had ECOG PS of 0–1. None of the patients had ECOG PS of 4. Two patients (4.0%) showed post-chemoradiotherapy recurrence of limited-stage SCLC, whereas the remainder had ES-SCLC. Brain and liver metastases were each observed in approximately one-third of patients, while bone metastases were observed in almost half of the patients. None of the patients with brain or bone metastases received palliative radiotherapy prior to starting first-line therapy. Superior vena cava syndrome was observed in 6% of cases; however, none of these patients had received palliative radiotherapy before initiating first-line therapy. Additionally, no patients underwent prophylactic cranial irradiation after first-line therapy.

**Table 1:** Patient characteristics.

Characteristics	All patients (n = 50)	Small total BTS (n = 43)	Large total BTS (n = 7)	*P*-value
Age (years)	72 (66–76)	72 (65–76)	74 (72–77)	0.39
<65 years	7 (14.0)	6 (14.0)	1 (14.3)	
≥65 years	43 (86.0)	37 (86.0)	6 (85.7)	
Sex				0.68
Male	33 (66.0)	29 (67.4)	4 (57.1)	
Female	17 (34.0)	14 (32.6)	3 (42.9)	
Smoking status				1.00
Never	3 (6.0)	3 (7.0)	0	
Current or former	47 (94.0)	40 (93.0)	7 (100.0)	
ECOG PS				0.046
0	12 (24.0)	12 (27.9)	0	
1	27 (54.0)	24 (55.8)	3 (42.9)	
2	10 (20.0)	6 (14.0)	4 (57.1)	
3	1 (2.0)	1 (2.3)	0	
4	0	0	0	
Stage				1.00
Post-treatment recurrence of LS	2 (4.0)	2 (4.7)	0	
ES	48 (96.0)	41 (95.4)	7 (100.0)	
Programmed death-ligand 1 inhibitors				1.00
Atezolizumab	43 (86.0)	37 (86.0)	6 (85.7)	
Durvalumab	7 (14.0)	6 (14.0)	1 (14.3)	
Brain metastasis				1.00
No	33 (66.0)	28 (65.1)	5 (71.4)	
Yes	17 (34.0)	15 (34.9)	2 (28.6)	
Liver metastasis				0.03
No	34 (68.0)	32 (74.4)	2 (28.6)	
Yes	16 (32.0)	11 (25.6)	5 (71.4)	
Bone metastasis				0.23
No	27 (54.0)	25 (58.1)	2 (28.6)	
Yes	23 (46.0)	18 (41.9)	5 (71.4)	
Superior vena cava syndrome				0.37
No	47 (94.0)	41 (95.3)	6 (85.7)	
Yes	3 (6.0)	2 (4.7)	1 (14.3)	
NLR	3.8 (2.6–5.2)	3.4 (2.5–5.0)	5.2 (2.9–13.7)	0.07
Low NLR (≤6.3)	43 (86.0)	39 (90.7)	4 (57.1)	
High NLR (>6.3)	7 (14.0)	4 (9.3)	3 (42.9)	
PLR	211.3 (171.2–324.7)	209.8 (153.2–319.6)	218.3 (176.5–507.7)	0.45
Low PLR (≤173.9)	15 (30.0)	14 (32.6)	1 (14.3)	
High PLR (>173.9)	35 (70.0)	29 (67.4)	6 (85.7)	
Total BTS	125.4 (99.3–166.1)	117.0 (90.0–156.0)	234.9 (195.7–254.0)	< 0.0001
Small total BTS (≤183.2 mm)	43 (86.0)	43 (100.0)	0	
Large total BTS (>183.2 mm)	7 (14.0)	0	7 (100.0)	

Data are presented as n (%) or medians (interquartile ranges). Fisher’s exact test was used to compare the proportions of categorical data between the groups. The Mann–Whitney *U*-test was used to compare the numerical data between the groups. BTS, baseline tumor size; ECOG PS, Eastern Cooperative Oncology Group Performance Status; ES, extensive-stage; IQR, interquartile range; LS, limited-stage; NLR, neutrophil-to-lymphocyte ratio; PLR, platelet-to-lymphocyte ratio

### 3.2 Cutoff values for the NLR, PLR, and total BTS

**Figure 1:**
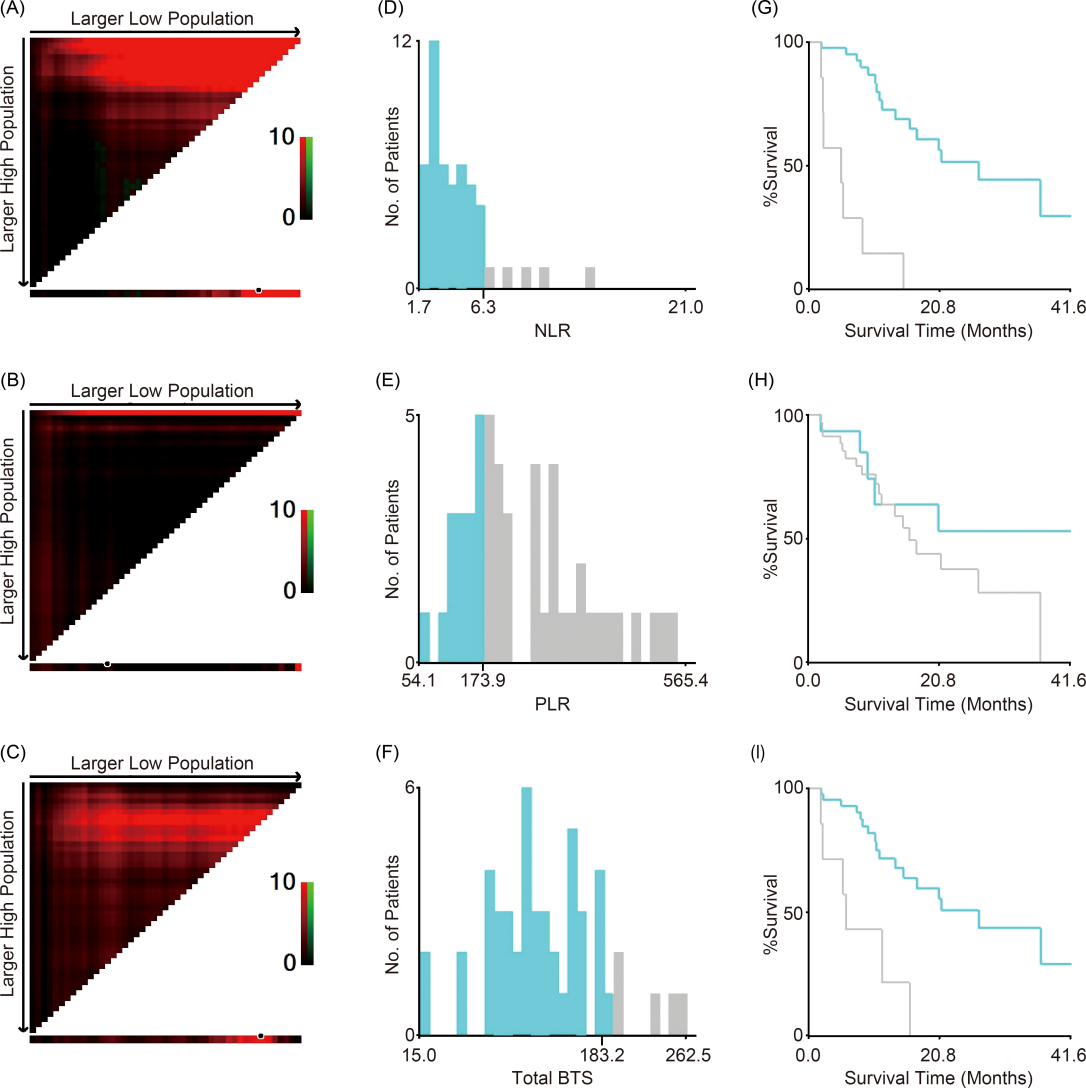
Cutoff values for NLR, PLR, and total BTS. X-tile software analyses were used to calculate the cutoff values. The optimal cutoff values for NLR, PLR, and total BTS for overall survival are indicated by black/white circles on the X-axes **(A–C)**; these values were 6.3 **(D)**, 173.9 **(E)**, and 183.2 **(F)**, respectively. Kaplan–Meier survival curves for overall survival of patients divided into two groups according to the optimal cutoff values are shown **(G–I)**. BTS, baseline tumor size; NLR, neutrophil-to-lymphocyte ratio; PLR, platelet-to-lymphocyte ratio.

Optimal cutoff values for the NLR, PLR, and total BTS for OS were calculated using X-tile software; these were 6.3, 173.9, and 183.2, respectively. The values are indicated by black/white circles on the X-axes in the images in **Figures 1A–C**. The count of cases for the groups below and above the optimal cutoff value is shown in the histograms in **Figures 1D–F**. Most patients had low NLR and total BTS but high PLR. Patients were categorized into two groups based on the optimal cutoff values for NLR, PLR, and total BTS, and their OS was analyzed using the Kaplan–Meier method (**Figures 1G–I**). More patients in the large total BTS group had poor ECOG PS and liver metastases compared to those in the small total BTS group (**Table 1**).

### 3.3 OS, PFS, and PPS for all patients and groups based on total BTS

**Figure 2:**
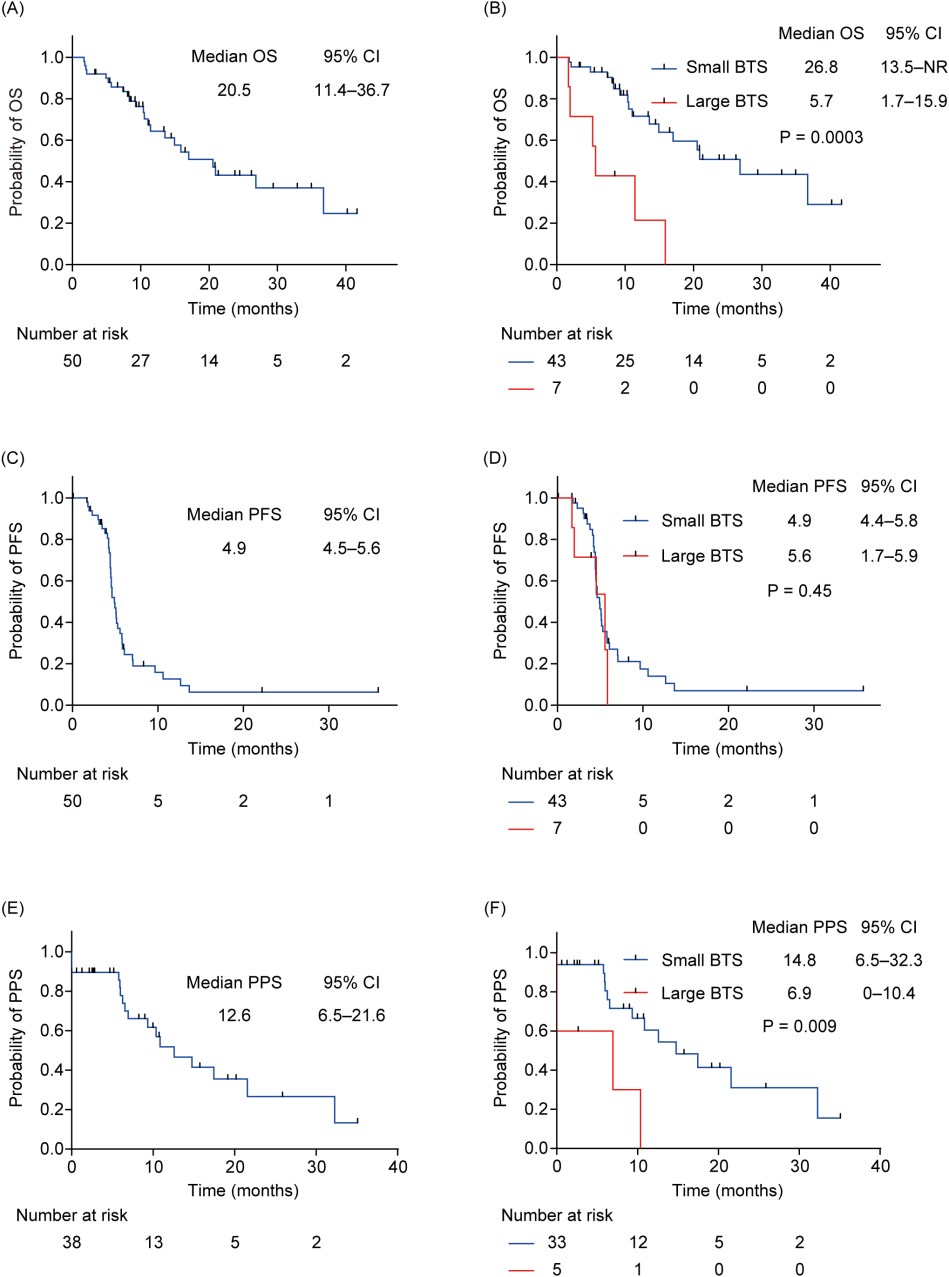
OS, PFS, and PPS for all patients and subgroups by total BTS. **(A)** Kaplan–Meier survival curves of the OS for all patients. **(B)** Kaplan–Meier survival curves of the OS of patients with small total BTS and that of those with large total BTS. **(C)** Kaplan–Meier survival curves of the PFS for all patients. **(D)** Kaplan–Meier survival curves of the PFS of patients with small total BTS and that of patients with large total BTS. **(E)** Kaplan–Meier survival curves of the PPS for all patients. **(F)** Kaplan–Meier survival curves of the PPS of patients with small total BTS and that of patients with a large total BTS. BTS, baseline tumor size; CI, confidential interval; NR, not reached; OS, overall survival; PFS, progression-free survival; PPS, post-progression survival.

The median observation period was 10.5 months (interquartile range: 7.4–20.9). At the data cutoff, 38 patients (76.0%) had reached PD, while 23 patients (46.0%) had died. The median OS of all patients was 20.5 months (95% CI: 11.4–36.7) (**Figure 2A**). The median OS of patients with small total BTS was 26.8 months (95% CI: 13.5–not reached), whereas that of patients with large total BTS was 5.7 months (95% CI: 1.7–15.9) (**Figure 2B**). A statistically significant difference in OS was noted between the two groups (*P* = 0.0003). The median PFS of all patients was 4.9 months (95% CI: 4.5–5.6) (**Figure 2C**). The median PFS of patients with small total BTS was 4.9 months (95% CI: 4.4–5.8), whereas that of patients with large total BTS was 5.6 months (95% CI: 1.7–5.9) (**Figure 2D**). There was no statistically significant difference in PFS between the two groups (*P* = 0.45). The PPS analysis included 38 patients whose first-line therapy reached PD (33 in the small total BTS group and five in the large total BTS group). The median PPS of all patients was 12.6 months (95% CI: 6.5–21.6) (**Figure 2E**). The median PPS of patients with small total BTS was 14.8 months (95% CI: 6.5–32.3), whereas that of patients with large total BTS was 6.9 months (95% CI: 0–10.4) (**Figure 2F**). A statistically significant difference in PPS was observed between the two groups (*P* = 0.009).

#### 3.3.1 Univariate and multivariate analyses of factors related to OS

**Figure 3:**
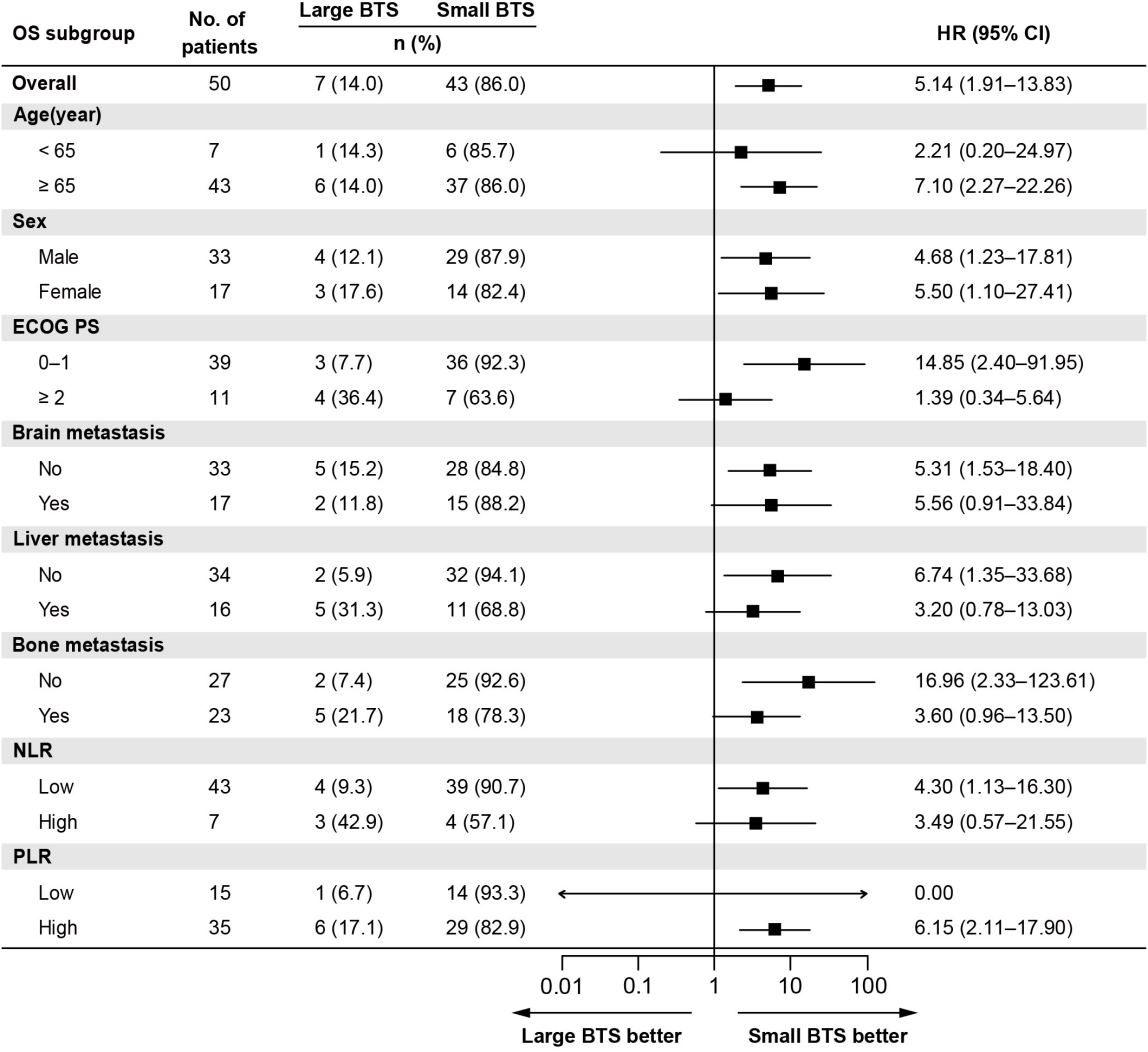
Subgroup analysis of the association between the total BTS and OS. BTS, baseline tumor size; CI, confidential interval; ECOG PS, Eastern Cooperative Oncology Group performance status; HR, hazard ratio; NLR, neutrophil-to-lymphocyte ratio; OS, overall survival; PLR, platelet-to-lymphocyte ratio.

**Table 2** shows the outcomes of univariate and multivariate analyses of factors related to OS. In the univariate Cox proportional hazard analysis, factors with *P <*0.05 were ECOG PS, NLR, and total BTS. Multivariate analysis showed that high NLR (HR: 15.35, 95% CI: 4.09–57.63, *P <*0.0001) and large total BTS (HR: 7.14, 95% CI: 1.89–26.96, *P* = 0.004) were significantly related to reduced OS. The association between total BTS and OS was analyzed in subgroups by baseline patient background. We identified a consistent trend for large total BTS to predict poor OS (**Figure 3**).

**Table 2:** Results of univariate and multivariate analyses of factors affecting overall survival (n = 50).

Variables	Category	Univariate analysis			Multivariate analysis		
		HR	95% CI	*P-*value	HR	95% CI	*P-*value
Age (years)	≥65 vs. <65	0.80	0.29–2.17	0.66	–	–	–
Sex	Female vs. Male	1.32	0.56–3.08	0.53	–	–	–
ECOG PS	≥2 vs. 0–1	2.97	1.24–7.11	0.01	0.51	0.14–1.92	0.32
Brain metastasis	Yes vs. No	0.62	0.24–1.59	0.32	–	–	–
Liver metastasis	Yes vs. No	1.96	0.82–4.66	0.13	–	–	–
Bone metastasis	Yes vs. No	1.26	0.54–2.94	0.59	–	–	–
NLR	High vs. Low	10.32	3.82–27.86	<0.0001	15.35	4.09–57.63	<0.0001
PLR	High vs. Low	1.85	0.67–5.06	0.23	–	–	–
Total BTS	Large vs. Small	5.14	1.91–13.83	0.001	7.14	1.89–26.96	0.004

BTS, baseline tumor size; CI, confidential interval; ECOG PS, Eastern Cooperative Oncology Group performance status; ES, extensive-stage; HR, hazard ratio; LS, limited-stage; NLR, neutrophil-lymphocyte ratio; PLR, platelet-lymphocyte ratio.

#### 3.3.2 Univariate analysis of factors related to PFS

Data from the univariate analyses of factors related to PFS are presented in **Table 3**. Univariate Cox proportional hazards analysis identified no factors with *P <*0.05. Therefore, multivariate analysis was not performed.

**Table 3:** Univariate analysis of factors affecting progression-free survival (n = 50).

Variables	Category	Univariate analysis		
		HR	95% CI	*P-*value
Age (years)	≥65 vs. <65	1.13	0.47–2.71	0.79
Sex	Female vs. Male	1.88	0.94–3.76	0.08
ECOG PS	≥2 vs. 0–1	1.57	0.67–3.65	0.30
Brain metastasis	Yes vs. No	0.48	0.23–1.02	0.06
Liver metastasis	Yes vs. No	1.19	0.59–2.41	0.63
Bone metastasis	Yes vs. No	1.66	0.86–3.18	0.13
NLR	High vs. Low	1.29	0.45–3.67	0.64
PLR	High vs. Low	1.23	0.59–2.56	0.58
Total BTS	Large vs. Small	1.44	0.55–3.75	0.46

BTS, baseline tumor size; CI, confidential interval; ECOG PS, Eastern Cooperative Oncology Group performance status; ES, extensive-stage; HR, hazard ratio; LS, limited-stage; NLR, neutrophil-to-lymphocyte ratio; PLR, platelet-to-lymphocyte ratio

#### 3.3.3 Univariate and multivariate analyses of factors related to PPS

**Table 4** shows the outcomes of univariate and multivariate analyses concerning factors related to PPS. In the univariate Cox proportional hazard analysis, factors demonstrating a significance level of *P <*0.05 were the ECOG PS, NLR, and total BTS. Multivariate Cox proportional hazards analysis identified no factors with a significance level of *P <*0.05.

**Table 4:** Results of univariate and multivariate analyses of factors affecting post-progression survival (n = 38).

Variables	Category	Univariate analysis			Multivariate analysis		
		HR	95% CI	*P-*value	HR	95% CI	*P-*value
Age (years)	≥65 vs. <65	1.03	0.33–3.18	0.97	–	–	–
Sex	Female vs. Male	0.97	0.36–2.65	0.96	–	–	–
ECOG PS	≥2 vs. 0–1	4.78	1.60–14.32	0.005	1.46	0.29–7.18	0.65
Brain metastasis	Yes vs. No	0.95	0.33–2.75	0.93	–	–	–
Liver metastasis	Yes vs. No	2.15	0.76–6.05	0.15	–	–	–
Bone metastasis	Yes vs. No	1.54	0.59–4.02	0.38	–	–	–
NLR	High vs. Low	5.82	1.76–19.24	0.004	4.12	0.86–19.76	0.08
PLR	High vs. Low	0.87	0.31–2.49	0.80	–	–	–
Total BTS	Large vs. Small	4.32	1.29–14.45	0.02	3.03	0.67–13.75	0.15

BTS, baseline tumor size; CI, confidential interval; ECOG PS, Eastern Cooperative Oncology Group performance status; ES, extensive-stage; HR, hazard ratio; LS, limited-stage; NLR, neutrophil-lymphocyte ratio; PLR, platelet-lymphocyte ratio.

### 3.4 Correlations between PFS-OS and PPS-OS

**Figure 4:**
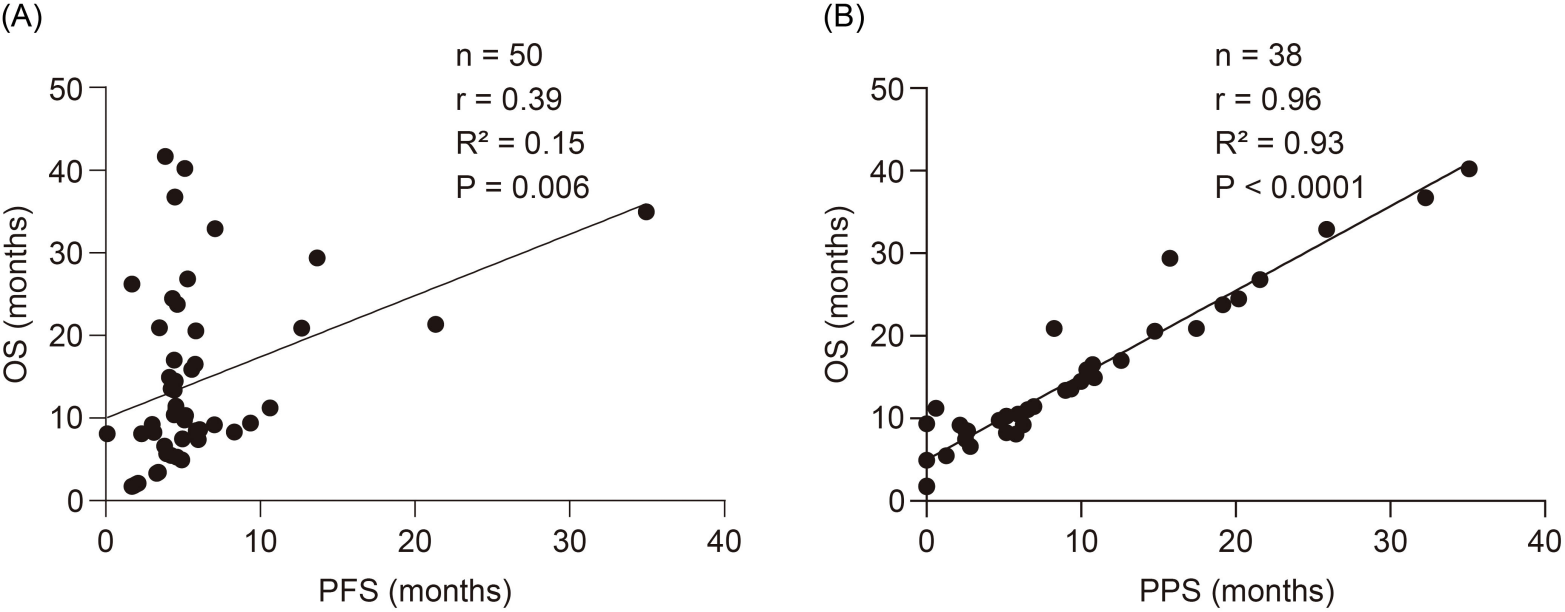
Correlations between the PFS-OS and the PPS-OS. **(A)** Correlation between the PFS and OS. **(B)** Correlation between the PPS and OS. OS, overall survival; PFS, progression-free survival; PPS, post-progression survival.

The correlations between PFS-OS and PPS-OS are presented in **Figures 4A, B**, respectively. According to the Spearman’s rank correlation coefficient and linear regression analysis, PPS was strongly correlated with OS (r = 0.96, R^2^ = 0.93, *P <*0.0001), whereas PFS was only moderately correlated with OS (r = 0.39, R^2^ = 0.15, *P* = 0.006).

### 3.5 Treatment response with first-line therapy

**Table 5** shows the treatment response with first-line therapy. The ORR and DCR for all patients were 66.0% and 92.0%, respectively. The difference in the ORR between the large and small total BTS groups was statistically significant, with the former having a lower ORR of 28.6% compared to the latter’s 72.1% (*P* = 0.03). The difference in the DCR between the two groups was not statistically significant, as their respective values were 95.3% and 71.4% (*P* = 0.09).

**Table 5:** Best overall response with first-line therapy stratified by total BTS.

Response	Total (n = 50)	Small total BTS (n = 43)	Large total BTS (n = 7)	*P-*value
Complete response (%)	4.0	4.7	0	
Partial response (%)	62.0	67.4	28.6	
Stable disease (%)	26.0	23.3	42.9	
Progressive disease (%)	0	0	0	
Not evaluable (%)	8.0	4.7	28.6	
Objective response rate (%)	66.0	72.1	28.6	0.03
Disease control rate (%)	92.0	95.3	71.4	0.09

BTS, baseline tumor size.

### 3.6 Adverse events with first-line therapy

**Table 6** shows the adverse events with first-line therapy. More than 70% of the patients in the small total BTS and large total BTS groups developed grade 3 or 4 neutropenia. An immune-related adverse event, type 1 diabetes, was observed in only two patients (4.7%) in the small total BTS group, while pneumonitis was observed in one patient (2.3%) in the small total BTS group and one (14.3%) in the large total BTS group.

**Table 6:** Adverse events with first-line therapy stratified by total BTS.

Event	Small total BTS group (n = 43)			Large total BTS group (n = 7)	
	Grade 1 or 2	Grade 3 or 4		Grade 1 or 2	Grade 3 or 4
			No. of patients (%)		
Any adverse event	18 (41.9)	33 (76.7)		2 (28.6)	5 (71.4)
Neutropenia	2 (4.7)	31 (72.1)		0	5 (71.4)
Febrile neutropenia	0	9 (20.9)		0	2 (28.6)
Thrombocytopenia	6 (14.0)	4 (9.3)		0	1 (14.3)
Anemia	2 (4.7)	4 (9.3)		1 (14.3)	2 (28.6)
Nausea	5 (11.6)	0		0	1 (14.3)
Fatigue	5 (11.6)	0		0	0
Decreased appetite	4 (9.3)	0		1 (14.3)	0
Type 1 diabetes	0	2 (4.7)		0	0
Pneumonitis	0	1 (2.3)		0	1 (14.3)
Hiccups	2 (4.7)	0		0	0
Increased creatinine	0	1 (2.3)		0	0
Sepsis	0	0		0	1 (14.3)
Constipation	1 (2.3)	0		0	0

BTS, baseline tumor size.

## 4 Discussion

The results of this study indicated that large total BTS and high NLR were independent negative predictors of OS in patients with ES-SCLC or post-chemoradiotherapy recurrence of limited-stage SCLC treated with chemotherapy plus PD-L1 inhibitor as first-line therapy. To our knowledge, no previous study has demonstrated that BTS is an independent predictor of OS in patients with SCLC undergoing chemotherapy plus PD-L1 inhibitor as first-line therapy.

The association between BTS and prognosis in the treatment with PD-1/PD-L1 inhibitors has not been previously reported in SCLC, but several reports have shown this relationship in other carcinomas and NSCLC. In a mouse model, subcutaneous inoculation of mice with melanoma cell lines followed by administration of PD-1 inhibitor has been shown to shrink small-size but not large-size tumors (33). Large BTS was an independent negative predictor of OS in patients with melanoma who received PD-1 inhibitor monotherapy (26) and of PFS and OS in patients with NSCLC who received PD-1/PD-L1 inhibitors monotherapy (24). In contrast, several studies have reported an association between BTS and prognosis in chemotherapy. In a mouse model, subcutaneous inoculation of mice with melanoma cell lines followed by highly potent cytotoxic chemotherapy reduced the size of small as well as large tumors (33). No association between BTS and PFS/OS was observed in patients with NSCLC who received platinum-based chemotherapy (24). Based on these reports, BTS is considered a characteristic prognostic factor for PD-1/PD-L1 inhibitors treatment. The negative effect of large BTS on the efficacy of PD-1/PD-L1 inhibitors has been reported as possibly being eliminated by adding chemotherapy, but this has not been conclusively validated (24). Our study suggested that BTS may be a prognostic factor in patients with SCLC who received chemotherapy plus PD-L1 inhibitor as first-line therapy. The ORR in the large total BTS group in this study was low, suggesting that lack of tumor shrinkage may have been one of the factors that hindered the efficacy of PD-L1 inhibitor. In recent years, a propensity score-matched multicenter retrospective analysis has reported that the addition of thoracic radiotherapy to chemotherapy and PD-L1 inhibitors improves ORR and OS in patients with ES-SCLC (34). As shown in our study, patients with a large total BTS have a poor prognosis, and adding thoracic radiotherapy to chemotherapy and PD-L1 inhibitors may improve the ORR and prolong OS. Thus, further research and reports are anticipated to explore these findings and their implications for clinical practice.

Several mechanisms by which large total BTS could be a poor prognostic factor in PD-1/PD-L1 inhibitors treatment have been reported. Suzuki et al. observed increased expression of genes related to NF-κB and Notch signaling pathways in patients with large total BTS based on immune-related gene expression profiling analysis of tumor tissue from patients with NSCLC before PD-1/PD-L1 inhibitors treatment (24). These signaling pathways do not inhibit the infiltration of anti-tumor immune cells into the tumor microenvironment. However, they may induce the infiltration of immunosuppressive M2-type macrophages and the formation of abnormal blood vessels, thereby rendering the tumors PD-1/PD-L1 inhibitors treatment-resistant (35–37). Alexander et al. reported that the ratio of circulating exhausted-phenotype-CD8 T-cell reactivation to pretreatment total BTS correlated with clinical outcome, based on peripheral blood immune profiling analysis of patients with melanoma before and after PD-1 inhibitor treatment. Lower values of this ratio were associated with shorter OS, implying a shorter OS in patients with larger pretreatment total BTS (38). Whether a similar mechanism is involved in patients with SCLC remains unknown; thus, further basic research is required in the future.

In our study, consistent with previous reports, we found that PPS was more strongly correlated with OS than PFS in patients with ES-SCLC receiving first-line therapy with chemotherapy plus PD-L1 inhibitors (29, 30). This observation indicates that PFS may not significantly influence OS in this treatment context. While OS and PPS were significantly shorter in the large total BTS group than in the small total BTS group, there was no significant difference in PFS between the two groups. This finding suggests that BTS is related to both OS and PPS, highlighting its potential importance as a prognostic factor. In the univariate analysis of factors related to PPS, BTS demonstrated significance with a *P*-value <0.05; however, it did not emerge as an independent predictor in the multivariate analysis. This discrepancy may arise from the fact that the PPS analysis was limited to patients whose first-line therapy resulted in PD, thereby reducing the sample size available for analysis.

In this study, a high NLR value was also an independent negative predictor of OS. Previous meta-analyses have indicated that a high NLR value before PD-1/PD-L1 inhibitors therapy negatively predicts PFS and OS in patients with NSCLC (39, 40). High NLR has been reported to be an independent negative predictor of PFS in patients with SCLC who received chemotherapy plus PD-L1 inhibitor as first-line treatment (17, 21). Our findings are consistent with the above reports and suggest that NLR may be a prognostic factor in patients with SCLC receiving chemotherapy plus PD-L1 inhibitor as first-line therapy. Although the biological and immunological mechanisms underlying this association are not fully understood, it has been reported that a decrease in lymphocytes involved in cancer immunity may indicate that cancer cells are in a state of immune escape (41). Moreover, neutrophils increase the secretion of inflammatory chemokines and cytokines, which may promote metastasis and tumor growth (42). Conversely, a neutrophil subset that favors anti-tumor immunity has recently been identified, while neutrophils have also been required for complete tumor eradication under PD-1 and cytotoxic T lymphocyte-associated antigen 4 inhibitors treatment (43, 44). Further studies are necessary to explore the impact of the NLR on tumor immunity.

In our study, we focused on clinically measurable factors as potential prognostic indicators. However, recent advancements in the classification of SCLC into four subtypes—based on transcription factor expression patterns and inflammation-related gene signatures—have opened new avenues for identifying patients who may derive greater benefit from PD-L1 inhibitors. The SCLC-inflammatory subtype is characterized by low expression of transcription factors, such as POU2F3, NEUROD1, and ASCL1, alongside high expression of genes associated with interferon-γ activation, human leukocyte antigen, and immune checkpoints, as well as increased immune cell infiltration. This subtype appears particularly responsive to PD-L1 inhibitors, suggesting that more targeted treatment strategies could improve outcomes for these patients (45). However, the subtyping process, which requires ribonucleic acid sequencing, poses challenges in routine clinical practice due to its complexity and resource requirements. Looking forward, integrating this subtype classification with easily measurable clinical parameters, such as BTS and NLR, may enable more accurate prognostic predictions for patients undergoing PD-L1 inhibitor therapy. This approach could help bridge the gap between advanced genomic classifications and practical clinical applications, ultimately leading to more personalized treatment strategies.

This study had some limitations. First, the sample size was relatively small. Second, the observation period was relatively short, potentially limiting the ability to capture long-term outcomes. However, despite these limitations, the identification of BTS as an independent negative predictor for OS is statistically significant. Third, there are potential limitations of the total BTS. Accurate evaluation of the total BTS is difficult in cases involving bone or pleural metastases. Moreover, in cases involving multiple small metastases falling under the category of unmeasurable disease, the total BTS may be judged to be small, although the prognosis is poor. Further optimization of the total BTS measurement method should be considered. Finally, the cutoff values for the NLR, PLR, and total BTS were calculated using X-tile software and may not have been optimal.

In conclusion, the findings of this study demonstrated that chemotherapy plus PD-L1 inhibitor in first-line therapy for patients with SCLC with large total BTS has a limited therapeutic effect. Clinicians routinely measure total BTS before chemotherapy induction and when determining treatment response. The method of measuring the total BTS is defined in the RECIST and is simple, non-invasive, and has little variation among measures. Therefore, the total BTS is easy to use in practice and may help predict prognosis when chemotherapy plus PD-L1 inhibitor is used as first-line therapy for patients with SCLC. Nevertheless, large-scale clinical trials and basic research are needed to consolidate this study’s results further.

## Data Availability

The raw data supporting the conclusions of this article will be made available by the authors, without undue reservation.
